# A new diagnostic sampling method in pure neural leprosy: the scraping of the myelin sheath^☆^^☆☆^

**DOI:** 10.1016/j.abd.2020.05.017

**Published:** 2021-03-20

**Authors:** Ilaria Trave, Alberto Cavalchini, Gianfranco Barabino, Aurora Parodi

**Affiliations:** a Section of Dermatology - Department of Health Sciences, University of Genoa, IRCCS - Ospedale Policlinico San Martino, Genoa, Italy; b Section of Dermatology- Ospedale Policlinico San Martino, Genoa, Italy

Dear Editor,

Pure Neural Leprosy (PNL) is a form of leprosy characterized by neural involvement without any skin lesions.^1^ PNL affects about 3%–10% of patients with leprosy and it can occur in any spectrum, although it is more frequent in the tuberculoid type.^2^ We present a case of a patient affected by PNL diagnosed through the scraping of myelin sheaths of the ulnar nerve, Ziehl-Neelsen (ZN) staining, and Polymerase Chain Reaction (PCR).

A 78-year-old man, a professional missioner in the Philippines and Papua New Guinea, has presented sensory loss (touch, pain, and temperature) of left foot and pain of left hand present over a period of 4 years. A physical examination revealed dorsal flexion deficit of the left foot, superficial paraesthesia, and dysesthesia of toes associated with impaired deep sensitivity. In addition, he presented paraesthesia and dysesthesia to the IV and V fingers of the left hand. The left ulnar nerve was palpable and enlarged on the left elbow and no cutaneous lesions were found. The research of Acid-Fast Bacillus (AFBs) in the nasal swab and the slit skin smears from earlobes and left elbow was negative. Motor and sensory action potential of the left ulnar nerve, left peroneal nerve, left anterior and posterior tibial nerves are suggestive of mono-neuritis multiplex. Magnetic Resonance Imaging (MRI) of the left elbow showed the enlarged ulnar nerve partially damaged by entrapment within the fibro-osseous tunnel. Neurosurgery allowed the debridement of the ulnar nerve and, at the same time, the scraping of the perineural tissue. ZN stain and PCR of the scraping were positive for the presence of *M. leprae* and the diagnosis of tuberculoid leprae with PNL was made. Antibodies against phenolic glycolipid-1 antigen (anti-PGL antibody) were negative. A therapy based on a combination of three drugs (rifampicin 600 mg once a month, dapsone 100 mg daily, and clofazimine 300 mg once a month and 50 mg daily) associated with prednisone 25 mg and gabapentin 300 mg (2 cp/die) was started with improvement of symptoms.

To the best of our knowledge, this is the first case of PNL diagnosed through scraping, ZN staining, and PCR test. Scraping is a technique that allows obtaining a clinical specimen rubbing a part of the body, in our case myelin sheaths of a nerve. The surface is scraped with a 15 Bard-Paker blade held at a right angle to the incision. Upon scraping, perineural tissue is obtained and examined by ZN staining and PCR test. Traditionally, diagnostic criteria for the diagnosis of PNL consist of nerve tissue samples obtained out of a nerve biopsy, analysis of PCR, and measure of anti-PGL-1 antibody levels.^3^ However, the invasive procedure of nerve biopsy was criticized by Abhishek De et al. because it has a high rate of complications.^4^ They proposed a simple technique of FNAC coupled with PCR in a pilot study^4^ and they confirmed its efficacy in a 4-year study.^5^ In our case, we did not use the technique of FNAC because an invasive procedure, surgery, was required to solve the compression of the ulnar nerve in the cubital tunnel. However, the scraping of the myelin sheath is a simple tissue sampling method during surgical procedures with less risk of nerve damage.

## Financial support

None declared.

## Authors’ contributions

Ilaria Trave: Conception and planning of the study; elaboration and writing of the manuscript; approval of the final version of the manuscript; obtaining, analyzing, and interpreting the data; effective participation in research orientation; intellectual participation in propaedeutic and/or therapeutic conduct of the cases studied; critical review of the literature; critical review of the manuscript.

Alberto Cavalchini: Conception and planning of the study; elaboration and writing of the manuscript; approval of the final version of the manuscript; obtaining, analyzing, and interpreting the data; effective participation in research orientation; intellectual participation in propaedeutic and/or therapeutic conduct of the cases studied; critical review of the literature; critical review of the manuscript.

Gianfranco Barabino: Conception and planning of the study; elaboration and writing of the manuscript; approval of the final version of the manuscript; effective participation in research orientation; intellectual participation in propaedeutic and/or therapeutic conduct of the cases studied; critical review of the literature; critical review of the manuscript.

Aurora Parodi: Conception and planning of the study; elaboration and writing of the manuscript; approval of the final version of the manuscript; obtaining, analyzing, and interpreting the data; effective participation in research orientation; intellectual participation in propaedeutic and/or therapeutic conduct of the cases studied; critical review of the literature; critical review of the manuscript.

## Conflicts of interest

None declared.
